# Sex chromosome evolution: historical insights and future perspectives

**DOI:** 10.1098/rspb.2016.2806

**Published:** 2017-05-03

**Authors:** Jessica K. Abbott, Anna K. Nordén, Bengt Hansson

**Affiliations:** Department of Biology, Lund University, Sölvegatan 37, 223 62 Lund, Sweden

**Keywords:** heteromorphic, homomorphic, degeneration, dosage compensation, turnover, timeline

## Abstract

Many separate-sexed organisms have sex chromosomes controlling sex determination. Sex chromosomes often have reduced recombination, specialized (frequently sex-specific) gene content, dosage compensation and heteromorphic size. Research on sex determination and sex chromosome evolution has increased over the past decade and is today a very active field. However, some areas within the field have not received as much attention as others. We therefore believe that a historic overview of key findings and empirical discoveries will put current thinking into context and help us better understand where to go next. Here, we present a timeline of important conceptual and analytical models, as well as empirical studies that have advanced the field and changed our understanding of the evolution of sex chromosomes. Finally, we highlight gaps in our knowledge so far and propose some specific areas within the field that we recommend a greater focus on in the future, including the role of ecology in sex chromosome evolution and new multilocus models of sex chromosome divergence.

## 1. Introduction

Many animals and some plants have sex chromosomes. In these species, sexual development is decided from a major sex-determining region [1], which triggers a cascade of sex-specific genes that control development into a male or female [2,3]. Old sex chromosomes have been extensively studied in mammals and *Drosophila* [3], and they are recognized by their specific features, including reduced recombination, degeneration, heteromorphic size and specialized, often sex-specific, gene content and expression [4]. Research on sex determination and sex chromosome evolution has increased over the past decade and is currently a dynamic field [1,2,5]. The study of sex chromosomes began in the late 1800s and early 1900s, when these special chromosomes were discovered [6], and today we have a good understanding of the general steps involved in sex chromosome evolution. However, some areas have not received as much attention as others, and we therefore aim to remedy this oversight by presenting a historical perspective on the development of sex chromosome evolution research. We provide an overview of important theories, models and empirical studies that have advanced the field and changed our understanding of sex chromosome evolution. Finally, we highlight gaps in our present knowledge and recommend an increased future focus on some specific areas within the field. We start with a brief outline of how sex chromosomes generally evolve.

## 2. Sex chromosome evolution

### (a) Genetic sex determination and recombination suppression

**Figure 1.**
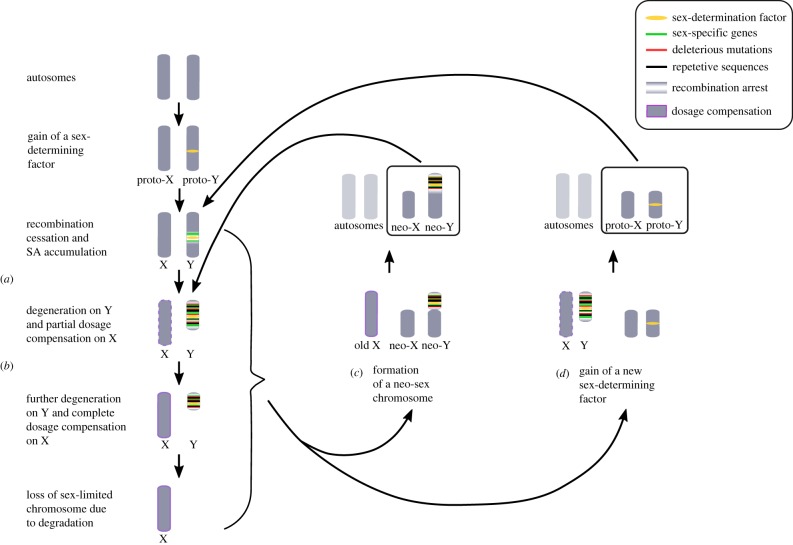
Overview of the dynamic evolution of sex chromosomes, illustrated in a male heterogametic system. Top left corner: an autosome pair in a hermaphrodite gains a sex-determining factor that evolves to become a highly heteromorphic pair of sex chromosomes, via cessation of recombination, degeneration (*a*) and evolution of dosage compensation (*b*). This progression can however be perturbed by a turnover event, such as the formation of a neo-sex chromosome (*c*) or a gain of a new sex-determining factor (*d*). In (*c*), the moderately degenerated Y chromosome fuses with an existing autosome, forming a new sex chromosome pair with an old sex-determining factor. In (*d*), an autosomal pair gains a new sex-determining factor, creating a completely new sex chromosome pair. The old Y is lost. In both (*c*) and (*d*), the old X may eventually gain diploidy through non-disjunction and subsequently lose dosage compensation, becoming an ordinary autosome pair. Figure adapted from [7]. Note that although (*c*) and (*d*) are shown as leading to chromosome turnovers, this progression is not inevitable. SA, sexually antagonistic allele.

The accepted theory of the evolution of heteromorphic sex chromosomes (figure 1) starts with a pair of homologous autosomes that gain a major sex-determining function through one or several genes [2,3,8]. This can happen in a system that already has a sex chromosome pair (and in that case it results in a so-called turnover, figure 1*c*,*d*) or in a hermaphrodite ancestor [2]. Two mutations are needed in order for separate sexes to evolve from hermaphroditism—one suppressing male fertility and the other suppressing female fertility, usually at different loci—otherwise a mixed mating system results (e.g. gynodioecy with females and hermaphrodites, which is the most common mixed system in plants) [8–10]. In case of a turnover, the new sex-determining gene needs to cause a fitness increase compared to the old sex-determining gene in order to invade [1].

Next, sex-specific genes become linked to the sex-determining region, and suppression of recombination evolves in the heterozygous sex since it is advantageous for these genes to be inherited together [2]. Recombination between the proto-X and proto-Y sex chromosomes (proto-Z and -W in female heterogametic systems) can be hindered either through gradual reduction with genetic modifiers or large inversions [8]. The recombination suppression region of the proto-sex chromosomes can expand further via the accumulation of sexually antagonistic genes (i.e. genes that are beneficial for one sex but detrimental for the other), near the sex-determining region [8,11].

### (b) Degeneration and dosage compensation

The increase of the non-recombining region results in strongly differentiated sex chromosomes, as genes decay via accumulation of deleterious mutations on the sex-limited Y chromosome [2,11]. Following Y degeneration (figure 1*a*), the homogametic sex (XX females) will have two copies of X-linked genes compared to the heterogametic sex's (XY males) one, resulting in unequal expression between the sexes. The solution is dosage compensation (figure 1*b*), which can be achieved in multiple ways (e.g. X chromosome inactivation in female mammals [12], or X hyperexpression in male *Drosophila* [12,13]). Dosage compensation is a common phenomenon taxonomically, but varies in its extent; it is almost complete in mammals, but is partial in birds and some snakes [12].

### (c) Sex chromosome turnovers

Though some organisms have lost the Y chromosome completely (e.g. crickets and dragonflies), not all sex chromosomes end up highly differentiated [1,2]. There are two main hypotheses: occasional recombination between X and Y due to sex-reversals and frequent turnover events. Sex chromosomes in sex-reversed female frogs (i.e. with an XY genotype) recombine as much as in XX-females, introducing new genetic variance on the Y [14]. However, this only works for species with relatively undifferentiated sex chromosomes—strongly differentiated sex chromosomes cannot recombine successfully [14]. Sex chromosome turnovers are very common in fishes and may result from the evolution of a new sex-determining gene on an autosome or transposition of a sex-determining locus to an autosome (figure 1*d*), or fusions between autosomes and existing sex chromosomes (formation of a neo-sex chromosome; figure 1*c*) [15].

### (d) Our changing views of sex chromosomes

Although most research has been carried out on highly heteromorphic sex chromosomes, we do know that sex chromosomes are diverse across living organisms, from the mammal XY and bird ZW to the less-studied haploid UV sex chromosomes (found in e.g. bryophytes [3,16,17]). We also know that there is a large variation in the level of degeneration of heteromorphic sex chromosomes, a variety of mechanisms of dosage compensation and a high frequency of sex chromosome turnovers in some groups but not others [1], making general patterns in sex chromosome evolution far from ‘general’ [1]. This is a relatively recent insight stemming from the explosion of sequencing technologies (see below) and suggests that our theories of sex chromosome evolution have likely been biased towards mammalian-style XY systems and shaped largely by studies of model organisms [18]. We, therefore, argue that a historic overview of key findings and empirical discoveries will put current thinking into context and help us better understand where to go next. To this end, we have compiled a timeline of sex chromosome evolution research (table 1), which illustrates the progress over time of our understanding of various stages in sex chromosome evolution. Although the points we include are inevitably somewhat subjective, we have attempted to cover all major discoveries in the evolution of sex chromosomes.

**Table 1.** A historical timeline of major theoretical and empirical advances in the study of sex chromosome evolution.

year	empirical advances	theoretical advances
Pre-1900	1845—haplodiploidy in honeybees proposed by Dzierzon [19] 1891—‘odd’ chromosomes discovered by Henking [6]	1880s—nutritional/metabolic theory of sex determination popular [20]
1900	1905—confirmation that the X is associated with sex by Stevens [21] 1905—discovery of the Y by Stevens [21] 1909—Morgan observes ZW and XO systems and demonstrates that variation in sex determination mechanisms is possible [22]	1902–1903—chromosomal theory of inheritance developed by Sutton [23] 1902—‘odd’ chromosomes suggested to be associated with sex by McClung [24] 1905—Wilson suggests that XO systems arise from XY systems [25] 1906—competing theories of sex determination: dose-dependence versus specific sex-linked factors [26,27] 1909—Castle suggests male-specific traits are located on the Y [28]
1910	1910—Morgan demonstrates sex linkage of white eyes in *Drosophila* [29] 1914—Bridges discovers XO males in *Drosophila* [30]	1914—Muller suggests restricted recombination between X and Y [31]
1920	1925—Bridges discovers XXY females in *Drosophila* [32] 1926—Morgan shows that XO *Drosophila* males are sterile [33]	1922—Haldane suggests that sex chromosomes evolve by the accumulation of many sex factors in tight linkage [34]
1930	1934—Koller and Darlington discover restricted recombination between the rat X and Y [35] 1939—Bridges shows that sex in *Drosophila* is determined by ratio of Xs to autosomes [36]	1931—sexual antagonism first proposed by Fisher [37] 1932—Muller and Painter point out that XY systems can be recessive X or dominant Y [38] 1933—Haldane argues that plants should have less Y degeneration than animals [39] 1935—Fisher calculates that X and Y should have similar numbers of lethals, but this is not consistent with data [40]
1940	1945—first description of UV chromosomes by Allen [41] 1946—rapid turnover of sex chromosome systems documented in platyfish by Gordon [42] 1949—first observation of inactivated X in mammals (Barr body) by Barr and Bertram [43] 1949—homomorphic sex chromosomes discussed by Matthey [44]	1947—existence of dosage compensation proposed by Muller, based on results in *Drosophila* [45]
1950	1952—Patterson and Stone find degeneration of autosomal fragments translocated to the *Drosophila* Y [46] 1957—Dobzhansky observes that the male X is twice as wide as the female X in *Drosophila*, consistent with dosage compensation via male hyperexpression [47] 1959—male determining factor on Y discovered in humans [48]	1958—Westergaard suggests that the evolution of dioecy in plants occurs by the evolution of tightly linked male and female sterility factors in concert with cessation of recombination between these factors [49]
1960	1961—Lyon demonstrates that females are genetic mosaics for the X in mice [50] 1964—different stages of sex chromosome evolution discovered in snakes by Beçak *et al.* [51] 1965—X-Y-W system of sex determination found in *Xiphophorus maculatus* by Kallman [52]	1965—Bowen suggests inversions can contribute to cessation of recombination on sex chromosomes [53] 1967—X and Y first proposed to have evolved from identical autosomes by Ohno [54] 1967—Hamilton develops selfish genetic element theory of Y degeneration [55] 1968—Frota-Pessoa and Aratangy develop inbreeding theory of Y degeneration [56] 1969—first model of suppression of recombination between sex chromosomes via sexual antagonism developed by Nei [57]
1970	1970—first evidence of sexually antagonistic fitness effects of an allele (colour genes in Poeciliids) by Kallman [58] 1978—dosage compensation in *Drosophila* is not via X inactivation, by Lucchesi [13] 1979—evidence of dosage compensation in *Caenorhabditis elegans* discovered by Duckett [59]	1970—Nei develops low population size model of degeneration of the Y [60] 1978—evolution of heteromorphic sex chromosomes modelled by Charlesworth and Charlesworth [10] 1978—Y chromosome evolution and dosage compensation modelled by Charlesworth [11] 1979—Bull develops theory for the origin of systems with uniparental males (haplodiploidy and paternal genome loss) [61]
1980	1982—homology between autosomal genes and Y-linked genes found (in humans, by Kunkel and Smith [62], in *Drosophila* by Steinemann [63])	1984—sex chromosomes proposed to be hotspots for sexual antagonism by Rice [64] 1987—model by Rice shows that sexual antagonism selects for cessation of recombination on sex chromosomes [65]
1990	1990—SRY discovered, proposed male ‘master gene’ in humans [66] 1992—first genetic map of human Y chromosome [67] 1994—Rice demonstrates degeneration of a non-recombining chromosome in real time [68] 1997—number of functional genes in non-recombining region of human Y increased from 8 to 20 by Lahn and Page [69]	1990s—debate over whether loss of the Y is inevitable in XY systems begins [18,70–73] 1999—‘evolutionary strata’ coined by Lahn and Page, first described on the human X [74]
2000	2003—full sequence of non-recombining region of human Y published (includes 27 protein-coding genes) [75] 2005—complete sequence of human X chromosome published [76]	2003—‘gene conversion’ proposed as mechanism preventing degeneration of Y by mimicking recombination [75] 2007—models of transitions between XY and ZW systems [77,78] 2009—Perrin models maintenance of homomorphic sex chromosomes via occasional recombination [14]
2010	2010—Lemos *et al.* find that Y polymorphism has functional consequences in *Drosophila* [79] 2012—Muyle *et al*. show that dosage compensation evolved rapidly in the young sex chromosomes of *Silene* [80] 2013—Vicoso and Bachtrog find reversal of a sex chromosome to an autosome in *Drosophila* [81]	2012—Jordan and Charlesworth find that sexual antagonism more likely in pseudo-autosomal region than on autosomes [82] 2014—hot potato model of sex chromosome turnover presented by Blaser *et al.* [83] 2014—Ùbeda *et al.* show that meiotic drive can help the spread of primitive sex chromosomes [4] 2015—Immler and Otto model evolution of UV systems [84]

## 3. History of key theory and empirical discoveries

### (a) Sex determination

At the end of the 1800s, the most popular theory for sex determination was nutritional/metabolic [20], since poor larval or maternal nutrition results in an overproduction of males in several species [85]. It was not until the early 1900s that the sex chromosomes were first associated with sex determination. Interestingly, early names for these chromosomes reflect this fact and describe other characteristics that made them unique (e.g. ‘odd’, accessory, idio- or heterotropic chromosomes) [18]. McClung first suggested in 1902 that ‘odd’ chromosomes (discovered by Henking in 1891 [6]) may be associated with sex [24]. He (incorrectly) proposed that the extra accessory (X) chromosome increased metabolism, indirectly causing the zygote to develop as a male.

Early work in *Drosophila* by Stevens and Morgan (reviewed in [18]) provided the empirical basis for the development of major new theories of sex determination by Stevens [26] and Wilson [27]. Stevens favoured the Mendelian view that one or a few specific factors on the X and Y determined sex. Wilson favoured an anti-Mendelian dose-dependent view—the higher the whole-X dose, the more the phenotype moves towards the female end of the spectrum. It is now clear that both theories are correct; some species have one or a few sex determination factors (figure 1), while others have polygenic sex determination [2]. Although we now know that sex in *Drosophila* is determined by the ratio of Xs to autosomes, a series of experiments in the early 1900s [28,33,36] cemented the idea that the X is associated with female traits, and the Y with male traits [18].

### (b) Sex chromosomes

Once the sex chromosomes were recognized as being intrinsic to sex determination (by the 1920s), specific theories of sex chromosome evolution could be developed (although Wilson suggested in 1905 [25] that XO systems likely evolve from XY systems). Surprisingly, it was initially assumed that the *Drosophila* and human XY chromosomes are homologous [18]. This is perhaps logical given that the first evidence of rapid sex chromosome turnover and rearrangements did not arrive until the 1940s and 1950s. Similarly, the first ZW systems were discovered quite early, but the first UV system was not described until 1945 and mixed XY and ZW systems within a single species were not discovered until the 1960s (table 1). This paints a picture of early conceptions of sex chromosomes as rather static and homogeneous entities.

### (c) Degeneration of Y

The first verbal theory of Y chromosome degeneration (figure 1*b*) was published by Muller in 1914 [31], but not really elaborated upon until the 1930s [38]. Haldane [39] suggested that plants should not experience Y chromosome degeneration to the same extent as animals due to pollen selection in the haplotypic phase. Fisher criticized the idea that the Y should degenerate via the accumulation of recessive lethals and predicted (incorrectly) that the X and Y should harbour equal numbers of lethal mutations [40]. In 1959, a male-determining factor was discovered on the human Y [48]. This discovery was important because it was previously assumed that sex was determined by the number of X chromosomes in mammals, with the Y just a non-functional fragment [18] (consistent with Bridges’ studies in *Drosophila* [30]).

Ohno [51] later suggested that the variation in sex chromosome morphology seen in snakes corresponds to different stages in the evolution of heteromorphic sex chromosomes, solidifying the idea that all Y chromosomes eventually degenerate. This changing view of the Y as potentially functional and evolutionarily labile sparked new theories of Y chromosome degeneration in the 1960s and 1970s and eventually led to a debate in the 1990s and 2000s over whether the human Y chromosome will eventually disappear [69,70]. The discovery of increasing numbers of protein-coding genes on the human Y [86], and the long-term stability of homomorphic sex chromosomes in some species [87,88] have changed our view of the Y (and W) as inevitably ‘born to be destroyed’ [89] (table 1).

### (d) Dosage compensation

Major advances in the 1940s and 1950s involved dosage compensation (figure 1*b*). The term was first coined in 1947 by Muller [45], just before the first empirical evidence of X inactivation in mammals in 1949 [43]. By the 1960s, it was clear that there are several types of dosage compensation, and evidence from chickens suggested that birds do not show evidence of chromosome-wide dosage compensation [51]. Charlesworth developed the first verbal model of the evolution of dosage compensation in 1978 [11], showing that as Muller's ratchet causes loss of functional genes on the Y, the X should evolve to compensate for this loss. Differences between taxa and evolutionary contingency were suggested to give rise to observed variation in forms of dosage compensation. Although there has been refinement of these early discoveries and theories (e.g. that dosage compensation on a gene-by-gene basis is the general pattern in most birds), later genomic and transcriptomic data have largely corroborated the idea that patterns of dosage compensation across taxa result from a combination of selection and contingency [12]. The largest advance has been the realization of just how diverse dosage compensation systems can be.

### (e) Modern theories of sex chromosome evolution

By the 1970s, all the pieces were in place for development of modern theories of sex chromosome evolution (table 1). It was known that sex chromosomes evolve from autosomes [54] via the cessation of recombination [34,57], leading to the evolution of heteromorphic sex chromosomes [6,22], dosage compensation [45] and the eventual degeneration of the Y (or W; [38]). This was followed by a burst of theory from the late 1960s to the 1980s, mainly focusing on the causes of Y chromosome degeneration and sexual antagonism as the selective agent favouring recombination suppression. The existence of sexually antagonistic loci in a broad sense (i.e. loci beneficial in one sex but not the other) was originally predicted by Fisher [37], and although early theories about the evolution of sex chromosomes implicitly assumed some sort of sex-specific advantage to recombination suppression [49], the role of sexual antagonism was not explicitly considered until Nei [57] (later expanded by Rice [65]).

Since the 1980s most theory has arguably been refinement of one of two very successful models of sex chromosome evolution. The first is Charlesworth and Charlesworth's [10] exploration of the evolution of sex chromosomes and separate sexes from an initially hermaphroditic state, and the second is Rice's [65] model of sexual antagonism favouring recombination suppression in systems with established sexes (e.g. in transitions from environmental sex determination to genetic sex determination, or other turnover events). Although sexual antagonism is not discussed as explicitly in Charlesworth and Charlesworth's model, it is still assumed to be an important factor selecting for recombination suppression. The main difference between these theories is, therefore, their starting point (hermaphroditic ancestor versus separated-sexed ancestor) rather than mechanism or subsequent evolutionary trajectory. Recent innovations generally focus on specific, previously uninvestigated aspects of sex chromosome evolution, such as the role of meiotic drive in the initial spread of sex-determining loci [4] or the evolution of UV systems [84] (table 1). Although UV systems were discovered rather early [41] and have been characterized in a number of species since [17], theory on UV systems has been surprisingly neglected (other than Bull's analysis of haploid dioecious sex chromosomes [90]). These advances therefore complement, not challenge, the established mechanisms described by Charlesworth and Charlesworth [10] and Rice [65].

In contrast to theory, advances in empirical data have been enormous since the 1990s thanks to the advent of genomic methods. Many of the processes proposed pre-1980 have now become testable in a range of organisms, and results are generally consistent with established theory. For example, the discovery of evolutionary strata on sex chromosomes [74,88] is consistent with block-wise recombination suppression via inversions, originally suggested in the 1950s [49]. A major advance has been the genomic characterization of sex chromosomes in various states of degeneration, demonstrating that degeneration of the Y (W) is not inevitable [88]. Other authors have recently reviewed this literature [1,5], so we will not dwell on it in detail here.

## 4. Where to now? Gaps in theory and empirical data

### (a) Ecological and experimental approaches

The field of sex chromosome evolution has naturally mainly been genetically driven, with the importance of ecology (e.g. local adaptation) and demography (e.g. range shifts) being poorly addressed [91]. Models often assume some level of sexual antagonism [65,77] and experiments verify that sexual antagonism is likely to be widespread [92], but the magnitude of sex-specific fitness variation under different ecological conditions is basically uncharacterised [93]. For example, locally adapted phenotypes (and underlying co-adapted gene complexes) may evolve in allopatry in different environments, e.g. small and large body size in poor and rich environments. If body size is sexually antagonistic, the adapted populations may be closer to the fitness optimum of one or the other sex [91]. If the populations stay separate, the locally adapted loci will be linked to male and female sex-determining regions equally often. However, if they become admixed, the probability that sexually antagonistic loci will be in linkage disequilibrium with sex-determining genes increases, fulfilling a key assumption of the sexual antagonism models. Similarly, a recent model found that X- and Z-linked genes play a particularly important role in local adaptation [94]. Consistent with this, Miura [95] found that hybridization events in the frog *Rana rugosa* can result in sex chromosome turnovers. We therefore recommend a greater focus on the role of ecology and demography in sex chromosome evolution.

It's currently unclear whether widespread sexual antagonism usually precedes the evolution of sex chromosomes or not [96], so one solution is to measure sexually antagonistic variation in species with a combination of sex determination systems, such as the snow skink (*Niveoscincus ocellatus*), where sex is temperature-dependent in the lowland but genetically determined in the highland [97]. Another possibility is experimental evolution, which has been successful in demonstrating other aspects of sex chromosome evolution, such as degeneration of a non-recombining chromosome (table 1, [68]). However, it may also give insight into whether a build-up of sexually antagonistic variation on the proto-sex chromosomes can happen in practice, by mimicking the evolution of a new sex-determining gene in a hermaphrodite, discussed in Abbott [98]. The evolution of dosage compensation could perhaps be investigated via artificially induced aneuploidy followed by experimental evolution.

### (b) The role of new technology

Sequencing technology is currently going deeper, and wider. Short-read sequencing has become increasingly affordable, leading to deeper coverage of genomes and transcriptomes. We believe that this will continue to impact research on sex chromosome evolution by broadening the taxonomical scope in studies aiming at understanding sex chromosome transitions and turnovers [83], and enabling studies of sex-biased genes with low expression. However, the most revolutionizing technological advancements are long-read sequencing techniques (e.g. single molecule real-time sequencing). These methods will improve genome assemblies in general, and in structurally difficult regions (e.g. Y and W) in particular [99], making it possible to test several hypotheses in a comparative framework, including the role of transposable elements and structural changes in sex chromosome evolution [8,58].

Furthermore, isoform sequencing of full-length transcripts will be able to shed new light on sex-specific exon use, and predictions regarding dosage compensation and gene silencing [100]. Finally, techniques such as chromosome conformation capture (e.g. Capture Hi-C) promise to impact our understanding of gene interactions and expression networks of autosomal and sex-linked genes [101,102], with implications for testing hypotheses of dosage compensation [11–13], and the role of sex chromosome–autosome interactions in adaptation and speciation [34]. We believe that data generated by these new technologies will both enable tests of (some) current hypotheses and lead to refinement and development of new theoretical frameworks.

### (c) Development of new theory

The field is overdue for development of fundamental new theory, since there have been relatively few major advances since the ground-breaking work of the 1970s and 1980s (table 1). One important arena is the role of ecology and demography in sex chromosome evolution, as discussed above. These phenomena are not well investigated theoretically, despite the recent development of exciting new models integrating sexual antagonism with demography [91], and the role of the sex chromosomes in local adaptation [94].

Second, a currently outstanding question is why only some sex chromosomes differentiate [9]. New technologies should help to resolve this problem, by producing data from a wider range of sex chromosome and sex determination systems. However, it also seems likely that as more taxa are investigated, additional complexities that are not consistent with existing theory will arise. Although the basic theories by Rice [65] and Charlesworth and Charlesworth [10] may not necessarily be supplanted, both were originally constructed with a rather narrow focus that is often forgotten today. It therefore seems likely that additional theory will be necessary to consolidate results from non-model organisms and help us to distinguish pattern and process. Although sexual antagonism and sex chromosomes are intimately related, cause and effect are difficult to determine and old sex chromosomes may be associated with different evolutionary processes from those on nascent sex chromosomes [2,12]. More theory on the links between pattern and process should help us in interpreting the results of data collection using new technologies.

Finally, we recommend an increased focus on multilocus models of sex chromosome evolution in future. Most models of sex chromosome evolution are built around one to a few loci [10,11,55–57,60,65], but results from the speciation literature suggest that multilocus models may show fundamentally different dynamics from few-locus models [103]. Flaxman *et al.* [103] found that genetic divergence between populations may evolve very suddenly once a tipping point in the number of selected loci and level of linkage disequilibrium between them has been reached. This is particularly interesting in the context of sex chromosome evolution, since the model did not assume any epistasis or cost of adaptation to a specific environment (i.e. sexual antagonism), which are typical ingredients in models of sex chromosome evolution [96]. We suggest that this framework can be extended to encompass sex chromosome evolution by treating males and females as different environments, making recombination rates between sex chromosomes equivalent to migration in the original model [103]. Should the results be similar, this would have interesting implications. For example, rapid divergence was facilitated by increased numbers of selected loci and low migration. This suggests that proto-sex chromosome size, intensity of selection for sexual dimorphism and intrinsic recombination rates would all be important parameters determining whether sex chromosomes undergo slow stepwise evolution, or rapid nonlinear divergence. These multilocus dynamics might then also influence the likelihood of evolving different forms of dosage compensation.

## 5. Conclusion: lessons from history

One interesting phenomenon that can be seen in table 1 is that our understanding of sex chromosome evolution—early characterization of XY or XO systems, followed by study of degeneration of the Y/W, mechanics of dosage compensation and finally the origin of heteromorphic sex chromosomes from autosomes or homomorphic sex chromosomes—has generally proceeded in reverse of the evolutionary process itself (figure 1). This means that many of the first discoveries of the properties of sex chromosomes have been in model organisms with highly diverged sex chromosomes such as *Drosophila* or humans, and this has of course biased our view of the diversity of sex chromosomes in nature [1]. A broader taxonomic focus incorporating many young sex chromosome systems [9] is now not only possible due to advances in sequencing, but it is also a natural next step in this progression. Moreover, ecological constraints and different types of sexual reproduction (and resulting sexual selection pressures) might influence the evolution of sex chromosomes, as discussed above. For example, if possession of a placenta increases the degree of sex-specific selection and therefore likelihood of sex chromosome differentiation, it could be interesting to look for an association between placental development and turnover events in fish.

A second observation is that although many of the processes involved in sex chromosome differentiation are similar to those discussed in the speciation literature (inversions, mutation accumulation, chromosomal rearrangements, etc.), the degree of crosstalk between the disciplines is somewhat limited. Early empirical studies found evidence of population variation in sex chromosomes [42,53], but a meta-population approach has rarely been applied to models of sex chromosome evolution [94]. In contrast, speciation research has arguably been more successful in attempting to tie together short-term ecological and demographic processes with long-term evolutionary patterns [104]. Approaches developed for the study of speciation could fruitfully serve as an inspiration for future research in sex chromosome evolution.

Finally, some authors have argued that the most important way forward is more comparative studies of different sex-determining systems [1,2,9]. Although we agree, it is important not to forget the contribution that manipulative experiments can make. Table 1 reveals rather few experimental manipulations [68,79], partly because manipulation of the sex chromosomes or sex determination is only possible in some systems. However a direct experimental test of the steps in sex chromosome evolution constitutes more robust evidence than a comparative study, especially considering the new manipulative possibilities opened up by the CRISPR/Cas9 system [105]. In any case, the field of sex chromosome evolution seems likely to remain active and dynamic for many years to come.
